# Gut microbiome causal impacts on the prognosis of breast cancer: a Mendelian randomization study

**DOI:** 10.1186/s12864-023-09608-7

**Published:** 2023-08-29

**Authors:** Weimin Hong, Guoxin Huang, Danhong Wang, Yadan Xu, Jie Qiu, Bin Pei, Da Qian, Xuli Meng

**Affiliations:** 1 General Surgery, Cancer Center, Department of Breast Surgery, Zhejiang Provincial People’s Hospital (Affiliated People’s Hospital), Hangzhou Medical College, Hangzhou, Zhejiang, 310000 China; 2 Department of Evidence-Based Medicine Center, Xiangyang No.1 People’s Hospital, Hubei University of Medicine, Xiangyang, 441000 China; 3 College of Pharmacy, Zhejiang University of Technology, Hangzhou, Zhejiang, 310014 China; 4 Department of Burn and Plastic Surgery-Hand Surgery, Changshu Hospital Affiliated to Soochow University Changshu No 1 People’s Hospital, Changshu, 215500 China

**Keywords:** Gut microbiome, Breast cancer, Oestrogen receptor status, Mendelian randomization study

## Abstract

**Background:**

Growing evidence has shown that gut microbiome composition is associated with breast cancer (BC), but the causality remains unknown. We aimed to investigate the link between BC prognosis and the gut microbiome at various oestrogen receptor (ER) statuses.

**Methods:**

We performed a genome-wide association study (GWAS) to analyse the gut microbiome of BC patients, the dataset for which was collected by the Breast Cancer Association Consortium (BCAC). The analysis was executed mainly via inverse variance weighting (IVW); the Mendelian randomization (MR) results were verified by heterogeneity tests, sensitivity analysis, and pleiotropy analysis.

**Results:**

Our findings identified nine causal relationships between the gut microbiome and total BC cases, with ten and nine causal relationships between the gut microbiome and ER-negative (ER-) and ER-positive (ER+) BC, respectively. The family Ruminococcaceae and genus *Parabacteroides* were most apparent among the three categories. Moreover, the genus *Desulfovibrio* was expressed in ER- BC and total BC, whereas the genera *Sellimonas*, *Adlercreutzia* and Rikenellaceae appeared in the relationship between ER + BC and total BC.

**Conclusion:**

Our MR inquiry confirmed that the gut microbiota is causally related to BC. This further explains the link between specific bacteria for prognosis of BC at different ER statuses. Considering that potential weak instrument bias impacts the findings and that the results are limited to European females due to data constraints, further validation is crucial.

**Supplementary Information:**

The online version contains supplementary material available at 10.1186/s12864-023-09608-7.

## Introduction

On a global scale, breast cancer (BC) is the most prevalent type of cancer affecting women [1]. Of concerning, accumulated data indicate that in 2020, there were 2.26 million new cases of BC [2]. Indeed, issues such as predisposition towards BC (family history), early start of the menstrual period, giving birth or occurrence of menopause at an advanced age, abnormal body weight, and exogenous hormone administration from oral contraceptives have a severe impact on the mortality rate of BC [3, 4]. Therefore, the high incidence and mortality rate of BC necessitates development of predictors for identifying BC patients. Moreover, it will assist doctors by improving precision medicine for BC.

It has gradually been accepted that BC is a complex disease with various aetiologies, development of which involves distinct entities with molecular and phenotypical backgrounds and different clinical results. Different types of BC have been categorized based on markers, such as oestrogen receptor (ER), basal or luminal expression profiles, and amplification of the human epidermal growth factor receptor 2 (HER2) gene. However, the prevalence of these cancers has not been elucidated profoundly [5].

In general, basal-like, HER2-positive, luminal-A-like, and luminal-B-like BC are the four intrinsic subtypes [6]. Their assessment is performed by immunohistochemical evaluation of expression of ER, progoesterone receptor (PR), HER2, and Ki-67 antigen [7]. In BC, ER plays a vital role, as 70% of detected cases show elevated ER expression [8]. Moreover, ER’s uniqueness differentiates it from other BC subtypes in terms of prognosis and biological characteristics, demonstrating sensitivity to antioestrogen therapy. The prognosis for BC patients with ER + status (defined as ER levels greater than or equal to 1%) is related to more favourable outcomes compared to those with ER- status (defined as ER levels less than 1%). ER has been regarded as an indicator with extreme importance in the prognosis of BC, as suggested by the guidelines of the two major networks, namely, the National Comprehensive Cancer Network (NCCN) and the European Society for Medical Oncology (ESMO) [9, 10]. Recently, there has been a growing interest in exploring the link between BC risk and the composition of the gut microbiota [11, 12]. However, the published information from relevant studies remains inadequate. Nevertheless, we did find evidence concerning a causal link between intestinal flora and other tumours [13, 14]. However, the same link has not been explored profoundly for BC. For example, in a study by Yang and colleagues, the authors discovered increased proliferation when colorectal cancer (CRC) cells were infected with *Fusobacterium nucleatum*. They observed an increase in the development of tumours in xenograft mouse models and CRC cell invasive activity [15].

Furthermore, Susan et al. found a direct interaction network between the intestinal microbiome and host immune cells that can promote cancer cell growth, also affecting cancer occurrence, development, and incidence [16]. Research has been conducted on the interaction between human microorganisms, especially the intestinal microbiome, and cancer [17]. Moreover, some results from epidemiological studies have shed light on the relationship between the human microbiome and health [18]. For example, Plaza-Diaz et al. conducted a retrospective study on the relationship between intestinal microbiome dysbacteriosis in the breast and the risk of BC [19].

In this context, Mendelian randomization (MR) is a popular technique for determining whether exposure and complicated results are causally related without any potentially detrimental intervention. By using genome-wide association study (GWAS) summary statistics, the MR technique was applied for exploration of a causal relationship between ER + and ER-BC risk and intestinal microbiota composition in this study.

## Materials and methods

### Research design

**Fig. 1:**
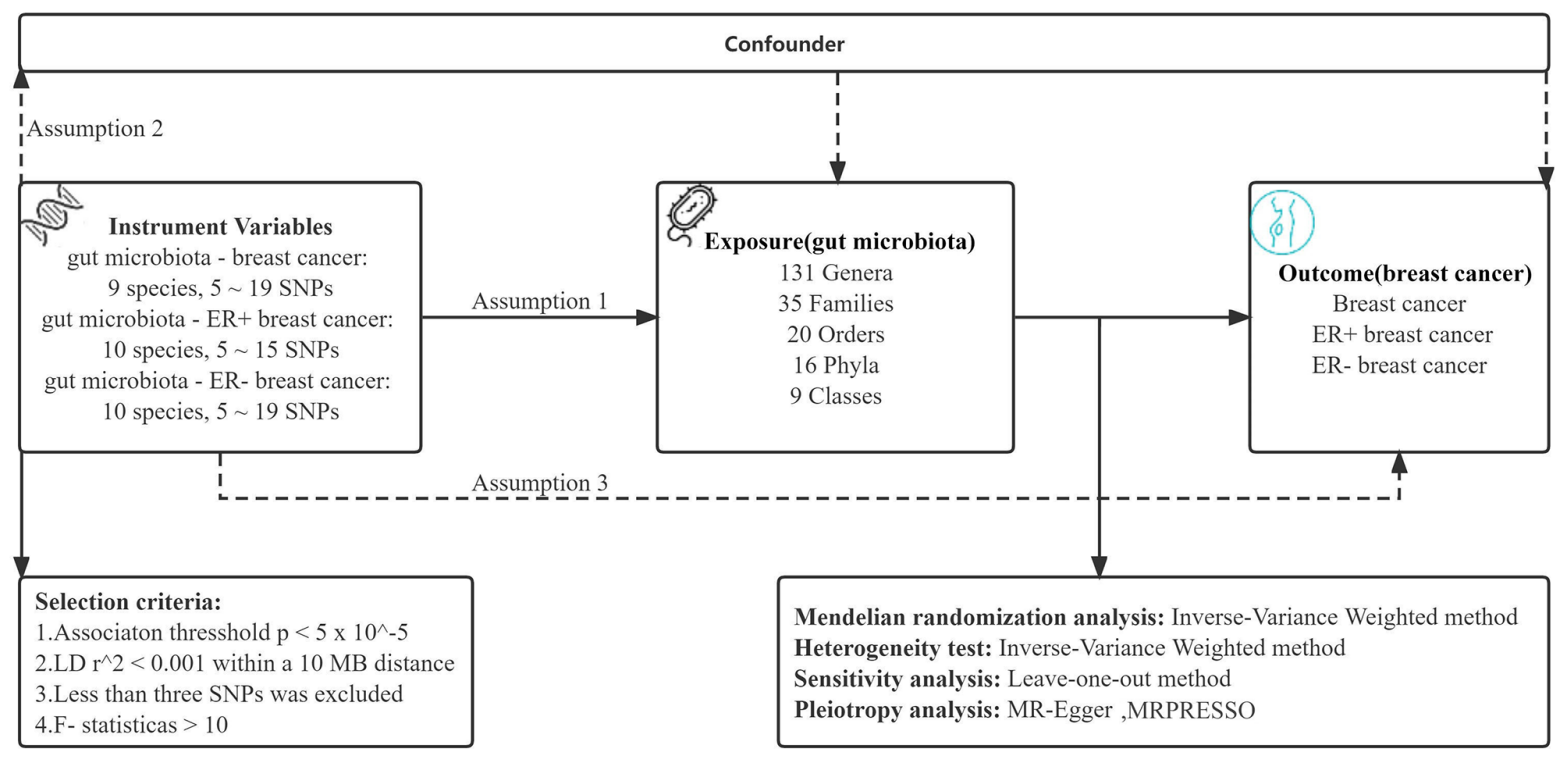
Schematic description of MR analysis in the discovery phase. Assumption 1: genetic variants are robustly associated with exposure. Assumption 2: genetic variants are not associated with confounders. Assumption 3: genetic variants affect outcomes only through the exposure of interest. LD, linkage disequilibrium

The causal relationship between gut microbial genera and the different statuses of ER BC was explored using a two-sample MR method. MR incorporates summary data from GWASs, minimizing the influence of confounding variables. Figure 1 illustrates the outline of the research design. Three critical assumptions were made to contend with the decrease in the impact of bias on the obtained results when using the MR technique. Initially, the intestinal microbiome should correlate strongly with instrumental variables (IVs), and the same IVs cannot be related to any potential confounders in later analyses. Last, the IVs should influence the outcome independently of exposure factors and other pathways [20].

### Database sources and tools

#### Human gut microbiota

Single-nucleotide polymorphisms (SNPs) related to the human gut microbiome composition were selected as IVs from the MiBioGen consortium (https://mibiogen.gcc.rug.nl/). The MiBioGen consortium contains 16 S RNA gene sequencing profiles and genotyping data from 18,340 samples, among which the correlation between a patient’s gut microbiota and genetic variation was studied [21]. The MiBioGen group collected data from 25 cohorts in 11 nations of European ancestry, including the exposure dataset, which had 122,110 SNPs of 211 taxa (from genus to phylum level). The selection criteria of IVs included the following: (1) association threshold setting of P < 5 × 10^− 5^ [22]; (2) performing a window of linkage disequilibrium (LD) for all IVs (r^2^ < 0.001, distance = 10 MB); (3) exclusion of SNPs that were less than three; and (4) an instrumental variable with mean F-statistics higher than 10 (F = R^2^ × (N − 2)/(1 − R^2^), where R^2^ represents the proportion of the variability of the exposure explained by each IV, and N means the sample size [23]).

#### Breast cancer

The outcome sources were the different ER statuses of BC, including total BC, ER + BC, and ER- BC. The Breast Cancer Association Consortium (BCAC) provided the BC data used [24], which comprised 10,680,257 SNPs and 228,951 samples (N = 122,977 cases and 105,974 controls). Of these, 127,442 samples (N = 21,468 cases; 105,974 controls) and 10,680,257 SNPs were related to ER- BC survival. On the other hand, 175,475 samples (N = 69,501 cases; 105,974 controls) and 10,680,257 SNPs were linked to ER + BC survival. The remaining three MR assumptions were satisfied by eligible IVs with a significance level of P < 5 × 10^− 5^, the specifics of which are detailed elsewhere. All information was retrieved from consortia providing publicly available summary statistics of European ancestry. Each employed GWAS received ethical approval from the relevant institutions.

### Statistical analysis

Inverse-variance weighted (IVW) is a method that combines multiple site effects of all SNPs when using MR analysis in a meta-analysis model. The premise of applying IVW is that all SNPs are valid IVs and completely independent of each other [25]. The IVW approach was primarily employed for varied ER statuses to calculate the causal link between gut microbiota and BC. We also performed the IVW method to examine the heterogeneity of each SNP (P<0.05) separately. The OR and 95% confidence interval (CI) represented the effect size. The leave-one-out strategy was applied in sensitivity analysis to validate the reliability of the data. Utilization of the MR‒Egger, MR pleiotropy residual sum and outlier (MR-PRESSO) tests (R package “MRPRESSO”) and intercept analysis in pleiotropy analysis confirmed the accuracy of the results obtained for the causal link of the gut microbiota to BC. At the same time, the Bonferroni approach was used to corrected P-values, in addition we provide the corrected p-values in Tables S1, S2 and S3 using the Benjamini-Hochberg (FDR) method. All statistical analyses were implemented using the “TwoSampleMR” package in R Version 4.2.2.

## Results

All raw results were listed in Table S1, S2 and S3 the manuscript, the overview of the relationship between gut microbiome and BC, ER- BC, and ER + BC were shown in Tables S1, S2 and S3, respectively.

### Causal effects of the gut microbiome on BC

The results provided in Table 1 from IVW analysis showed that genus *Parabacteroides* ID 954 (odds ratio (OR) = 0.87, 95% CI, 0.79–0.96, P = 0.007), genus *Adlercreutzia* ID 812 (OR = 0.92, 95% CI, 0.87–0.98, P = 0.014), genus *Ruminococcaceae* UCG-013 ID 11,370 (OR = 0.92, 95% CI, 0.86–0.99, P = 0.027), genus *Lactobacillales* ID 1800 (OR = 0.94, 95% CI, 0.88-1.00, P = 0.041), genus *Desulfovibrio* ID 3173 (OR = 0.94, 95% CI, 0.88-1.00, P = 0.043), genus *Ruminiclostridium* 6 ID 11,356 (OR = 0.94, 95% CI, 0.89-1.00, P = 0.046) and family Rikenellaceae ID 967 (OR = 0.92, 95% CI, 0.85-1.00, P = 0.047) acted as preventative measures for total BC. Alternatively, the genera *Ruminococcaceae* ID 2050 (OR = 1.11, 95% CI, 1.00-1.22, P = 0.035) and *Sellimonas* ID 14,369 (OR = 1.05, 95% CI, 1.01–1.09, P = 0.013) appeared to be linked to a greater risk of total BC (Table 1).

**Table 1:** MR estimates for the association between the gut microbiota and BC

Exposure	Outcome	SNPs	OR (95% CI)	P Effect	P Heterogeneity	P Intercept	R^2 Val	F Val	P Bonferroni	Direction
genus *Parabacteroides* id.954	Breast cancer	5	0.87 (0.79–0.96)	0.01	0.78/0.32	0.74	0.01	21.57	> 3.8e-4	Positive
genus *Sellimonas* id.14,369		9	1.05 (1.01–1.09)	0.01	0.37/0.47	0.75	0.02	21.99	> 3.8e-4	Negative
genus *Adlercreutzia* id.812		8	0.92 (0.87–0.98)	0.01	0.44/0.16	0.22	0.02	27.27	> 3.8e-4	Positive
genus *Ruminococcaceae* UCG013 id.11,370		11	0.92 (0.86–0.99)	0.03	0.85/0.75	0.83	0.02	21.36	> 3.8e-4	Positive
family Ruminococcaceae id.2050		9	1.11 (1.01–1.22)	0.04	0.13/0.28	0.11	0.02	21.01	> 3.8e-4	Negative
order *Lactobacillales* id.1800		14	0.94 (0.88-1.00)	0.04	0.70/0.75	0.39	0.03	21.93	> 2.5e-3	Positive
genus *Desulfovibrio* id.3173		10	0.94 (0.88-1.00)	0.04	0.29/0.36	0.22	0.02	21.40	> 3.8e-4	Positive
genus *Ruminiclostridium6* id.11,356		16	0.94 (0.89-1.00)	0.05	0.27/0.12	0.20	0.03	21.19	> 3.8e-4	Positive
family Rikenellaceae id.967		19	0.92 (0.85-1.00)	0.05	0.01/0.43	0.54	0.03	21.48	> 1.4e-3	Positive

SNP: single-nucleotide polymorphism, OR: odds ratio (OR = 1 exposure does not affect odds of the outcome, OR > 1 exposure associated with higher odds of the outcome, OR < 1 exposure associated with lower odds of the outcome), P heterogeneity: MR‒Egger method/MRPRESSO method

### Gut microbiome causal impacts on ER- BC

Table 1 provides evidence suggesting that the genus *Desulfovibrio* ID 3173 (OR = 0.84, 95% CI, 0.75–0.93, P = 0.001, IVW method), genus *Eubacterium Xylanophilum* Group ID 14,375 (OR = 0.82, 95% CI, 0.71–0.95, P = 0.007), genus *Lachnospiraceae* NK4A136 Group ID 11,319 (OR = 0.88, 95% CI, 0.79–0.97, P = 0.010), genus *Dorea* ID 1997 (OR = 0.84, 95% CI, 0.73–0.97, P = 0.014), genus *Clostridium sensustricto1* ID 1873 (OR = 0.86, 95% CI, 0.75–0.99, P = 0.033), genus *Parabacteroides* ID 954 (OR = 0.82, 95% CI, 0.68–0.98, P = 0.034), genus *Olsenella* ID 822 (OR = 0.93, 95% CI, 0.87–0.99, P = 0.034) and genus *Candidatus Soleaferrea* ID 11,350 (OR = 0.91, 95% CI, 0.83-1.00, P = 0.040) reduced the risk of ER- BC. On the other hand, the family Ruminococcaceae ID 2050 (OR = 1.15, 95% CI, 1.00-1.33, P = 0.049) and unknown genus ID 826 (OR = 1.13, 95% CI, 1.01–1.26, P = 0.027) presented a risk for the development of ER- BC (Table 2).

**Table 2:** MR estimates for the association between the gut microbiota and ER- BC

Exposure	Outcome	SNPs	OR (95% CI)	P Effect	P Heterogeneity	P Intercept	R^2 Val	F Val	P Bonferroni	Direction
family Ruminococcaceae id.2050	ER- Breast cancer	9	1.15 (1.00-1.33)	0.05	0.97/0.97	0.34	0.02	21.01	> 1.4e-3	Negative
genus *Candidatus Soleaferrea* id.11,350		10	0.91 (0.83-1.00)	0.04	0.34/0.49	0.18	0.02	21.37	> 3.8e-4	Positive
genus *Clostridium sensustricto1* id.1873		6	0.86 (0.75–0.99)	0.03	0.47/0.65	0.45	0.01	20.48	> 3.8e-4	Positive
genus *Desulfovibrio* id.3173		10	0.84 (0.73–0.93)	0.00	0.46/0.49	0.42	0.02	21.40	> 3.8e-4	Positive
genus *Dorea* id.1997		10	0.84 (0.73–0.97)	0.01	0.78/0.65	0.76	0.02	21.07	> 3.8e-4	Positive
genus *Eubacterium xylanophilum* group id.14,375		9	0.82 (0.71–0.95)	0.01	0.30/0.45	0.81	0.02	21.67	> 3.8e-4	Positive
genus *Lachnospiraceae* NK4A136 group id.11,319		15	0.88 (0.79–0.97)	0.01	0.52/0.63	0.80	0.02	21.45	> 3.8e-4	Positive
genus *Olsenella* id.822		11	0.93 (0.87–0.99)	0.03	0.43/0.45	0.06	0.02	21.40	> 3.8e-4	Positive
genus *Parabacteroides* id.954		5	0.82 (0.68–0.98)	0.03	0.41/0.06	0.79	0.01	21.57	> 3.8e-4	Positive
unknown genus id.826		14	1.13 (1.01–1.26)	0.03	0.34/0.30	0.39	0.02	21.30	> 3.8e-4	Negative

ER: oestrogen receptor, SNP: single-nucleotide polymorphism, OR: odds ratio (OR = 1 exposure does not affect odds of the outcome, OR > 1 exposure associated with higher odds of the outcome, OR < 1 exposure associated with lower odds of the outcome), P heterogeneity: MR‒Egger method/MRPRESSO method

### Causal effects of the gut microbiome on ER + BC

Using the IVW method, we discovered that the genus *Adlercreutzia* ID 812 (OR = 0.88, 95% CI, 0.81-0.95, P = 0.001), genus *Parabacteroides* ID 954 (OR = 0.87, 95% CI, 0.77–0.98, P = 0.024), *Marvinbryantia* ID 2005 (OR = 0.92, 95% CI, 0.85–0.99, P = 0.039) and family Streptococcaceae ID 1850 (OR = 0.92, 95% CI, 0.84-1.00, P = 0.042), as well as family Rikenellaceae ID 967 (OR = 0.91, 95% CI, 0.83–0.99, P = 0.029), were linked to a decreased incidence of ER + BC (Table 3). Conversely, the genus *Sellimonas* ID 14,369 (OR = 1.08, 95% CI, 1.03–1.13, P = 0.001), genus *Ruminococcus Gauvreauii* Group ID 11,342 (OR = 1.11, 95% CI, 1.01–1.20, P = 0.022), family Ruminococcaceae ID 2050 (OR = 1.15, 95% CI, 1.04–1.26, P = 0.005), and order Bacillales ID 1674 (OR = 1.05, 95% CI, 1.00-1.10, P = 0.042) were linked to a high risk of ER + BC (Table 3). We did not find significant correlations between the other taxa, 211 in number, and BC. Of note, when applying the Bonferroni method to adjust for multiple comparisons across various classification levels, none of the results in this study achieved a significance level that survived the Bonferroni-corrected threshold. Moreover, considering that Bonferroni correction may lead to false-negatives, the results of this study were not subjected to correction.

**Table 3:** MR estimates for the association between the gut microbiota and ER + BC

Exposure	Outcome	SNPs	OR (95% CI)	P Effect	P Heterogeneity	P Intercept	R^2 Val	F Val	P Bonferroni	Direction
family Rikenellaceae id.967	ER + Breast cancer	19	0.91 (0.83–0.99)	0.03	0.06/0.05	0.06	0.03	21.48	> 1.4e-3	Positive
family Ruminococcaceae id.2050		9	1.15 (1.04–1.26)	0.01	0.36/0.56	0.36	0.02	21.01	> 1.4e-3	Negative
family Streptococcaceae id.1850		12	0.92 (0.94-1.00)	0.04	0.78/0.76	0.78	0.02	21.57	> 1.4e-3	Positive
genus *Adlercreutzia* id.812		8	0.88 (0.81–0.95)	0.00	0.47/0.45	0.47	0.02	21.27	> 3.8e-4	Positive
genus *Marvinbryantia* id.2005		10	0.92 (0.85–0.99)	0.03	0.69/0.56	0.69	0.02	21.81	> 3.8e-4	Positive
genus *Parabacteroides* id.954		5	0.87 (0.77–0.98)	0.02	0.50/0.14	0.05	0.01	21.57	> 3.8e-4	Positive
genus *Ruminococcus gauvreauii* group id.11,342		11	1.11 (1.01–1.20)	0.02	0.36/0.35	0.36	0.02	21.84	> 3.8e-4	Negative
genus *Sellimonas* id.14,369		9	1.08 (1.03–1.13)	0.00	0.37/0.37	0.37	0.02	21.99	> 3.8e-4	Negative
order *Bacillales* id.1674		8	1.05 (1.00-1.10)	0.04	0.40/0.14	0.40	0.02	21.55	> 2.5e-3	Negative

ER: oestrogen receptor, SNP: single-nucleotide polymorphism, OR: odds ratio (OR = 1 exposure does not affect odds of the outcome, OR > 1 exposure associated with higher odds of the outcome, OR < 1 exposure associated with lower odds of the outcome), P heterogeneity: MR‒Egger method/MRPRESSO method

### Sensitivity analysis

The accuracy of the results between the gut microbiome and different statuses of ER of BC were evaluated by sensitivity analysis. No significant heterogeneity was identified (Tables 1, 2 and 3). Among the bacteria, the MR‒Egger and MR-PRESSO intercept tests showed no evidence of pleiotropy (P > 0.05) (Tables 1, 2 and 3). The F value was greater than 20, indicating no weak IV bias (Table 1, 2 and 3). Moreover, the MR estimation results predicted by leave-one-out analysis were not driven by specific SNPs.

## Discussion

In this two-sample MR study, we identified nine causal relationships between the gut microbiome and total BC, nine between the gut microbiome and ER + BC, and ten between the gut microbiome and ER- BC. We also discovered that the family Ruminococcaceae and genus *Parabacteroides* appeared in three categories, while the genus *Desulfovibrio* was proven to participate in the link between total BC and ER- BC. Concerning the association between ER + BC and total BC, the genera *Sellimonas* and *Adlercreutzia* and family Rikenellaceae exhibited significant implications. These findings provide insight into the clinical and experimental investigation of bacterial targets.

### BC

The analysis showcased causal relationships between the genus *Parabacteroides*, genus *Sellimonas*, genus *Adlercreutzia*, genus *Ruminococcaceae* UCG-013 within this family, genus *Desulfovibrio*, genus *Ruminiclostridium* 6, order Lactobacillales, family Rikenellaceae and total BC. Of note, when we set the threshold for statistical significance at a p value of 5 × 10^− 8^, no causal relationship between gut microbiota and the three types of BC was identified. Subsequently, we applied Bonferroni correction to account for multiple comparisons at different taxonomic ranks, with varying adjusted p values (adjusted p value < 3.1 × 10^–3^ for phylum, adjusted p value < 5.6 × 10^–3^ for class, adjusted p value < 2.5 × 10^–3^ for order, adjusted p value < 1.4 × 10^–3^ for family, and adjusted p value < 3.8 × 10^–4^ for genus), considering the number of bacterial traits within each specific gut microbiota rank. However, after applying this correction, none of the results in our study reached the adjusted p value threshold (Table S4). As a result, we did not perform correction on our findings. It is worth noting that application of Bonferroni correction may lead to false-negatives. This can be attributed to the complex interplay typically observed in the gut-cancer axis, which is often influenced by multiple factors. Furthermore, the individual contribution of a single microbiota genus in causing disease may not hold as significant a role as previously hypothesized.

There is limited research on the relationship between *Adlercreutzia* and BC. A previous study showed that stromal tissue of the breast had a high percentage of fat and a low percentage of fibrosis in malignant versus benign breast disease. At the same time, *Adlercreutzia* (Actinobacteria) exhibited a positive association with fibrosis percentage at the order level [26]. *Adlercreutzia* was also found to have an increased abundance after all dietary treatment groups in which those nutritional compounds showed an inhibiting effect on tumour growth [27]. Both studies agree with our findings.

Surprisingly, Lactobacillales was mainly studied in animal experiments in which the research focused on BC. A laboratory trial showed that giving milk fermented by *Lactobacillus casei* CRL431 (belonging to order Lactobacillales) maintained improved anticancer response, decreased tumour development, and reduced lung metastases and tumour vascularity in mice [28]. In another study, the authors gave the same application to mice and had similar results [29], thus supporting our results.

Using 16 S rRNA sequencing to compare the gut microbiome between individuals with nonpuerperal mastitis and healthy individuals, the outcomes demonstrated a positive correlation between the family Ruminococcaceae in breast tissue and differential expression of immune-related genes [30]. However, Flores et al. examined the level of nonovarian systemic oestrogens, which might contribute to higher BC risk in postmenopausal women. They found a strong association with the faecal Ruminococcaceae family [31]. Another finding suggested that *Desulfovibrio* might be used as a diagnostic marker because premenopausal BC patients have a decreased abundance of short-chain fatty acid-producing bacteria compared to healthy premenopausal women [32]. The research suggests that the family Ruminococcaceae and genus *Desulfovibrio* separately play a protective role in nonpuerperal mastitis patients and postmenopausal BC patients, which was consistent with our overall study findings. Contrary to our results, the family Ruminococcaceae presented a risk for postmenopausal women. Its function might be related to women with BC at different times.

However, no relevant study has investigated the relationship between *Parabacteroides*, *Sellimonas*, *Ruminiclostridium* 6, Rikenellaceae, and total BC.

### ER- BC

We further found evidence of causal relationships between the genus *Desulfovibrio*, genus *Eubacterium Xylanophilum* Group, genus *Lachnospiraceae* NK4A136 Group, genus *Dorea*, unknown genus, genus *Clostridium sensustricto1*, genus *Parabacteroides*, genus *Olsenella*, genus *Candidatus Soleaferrea*, and family Ruminococcaceae with ER-BC.

Recently, authors have reported the categorization of patients with HER2 + BC who received trastuzumab into nonresponsive (NR) and complete response (R) groups. Compared to R patients, NR patients had lower α-diversity and lower abundance of Lachnospiraceae. Additionally, transfer of faecal microbiota into HER2 + BC mice from NR and R recapitulated the response to trastuzumab observed in patients [33]. An interesting study also showed that BC survivors had a higher risk of cancer recurrence with a lower relative abundance of Lachnospiraceae [34]. The corresponding research showed that Lachnospiraceae can serve as a protective factor against BC.

A clinical trial on BC survivors sequencing faecal microbes demonstrated an abundance of *Dorea*, which was negatively associated with physical functioning, vitality, and mental health subscales. Alternatively, BC survivors without obesity had a significantly higher relative abundance of the genus *Ruminococcus* (belonging to the family Ruminococcacea) [35]. The study indicated that *Dorea* and *Ruminococcus* were linked to BC risk, though our results did not indicate this for the former. However, *Dorea* and uncultured *Ruminococcus*, which were considered to be signature bacteria to distinguish neoadjuvant chemotherapy (NAC) effectual group patients from the NAC noneffectual group, were increased in the NAC effectual group, according to analysis of the relationship between the gut microbiome and BC patients’ responses to NAC efficacy [36]. This diversity suggests that more in-depth investigation is warranted. It also suggests that the intestinal flora may be a target for therapy in different BC subtypes. Interestingly, an unknown genus, 826, acted as a precursor to the development of ER-BC, though more research on its biology is needed. We did not find evidence for a genetic correlation between other members of the gut microbiome and ER- BC.

### ER + BC

Our findings suggested that the genera *Adlercreutzia*, *Sellimona*, *Ruminococcus gauvreauii* group, *Parabacteroides*, and *Marvinbryantia*; the families Ruminococcacea, Rikenellaceae, and Streptococcaceae; and the order Bacillales had a causal relationship with ER + BC.

Notably, in a clinical study on postmenopausal women with ER + BC and 153 faecal samples, a significantly lower richness of *Marvinbryantia*, *Ruminococcaceae* NK4A214, and *Ruminococcaceae* UCG-005 was found after treatment [37]. The results for Ruminococcaceae agree with ours, yet those for *Marvinbryantia* do not.

Unfortunately, there is a great lack of data exploring the relationship between other intestinal bacterial genera and ER + BC. The order Bacillales exhibited a positive association with fibrosis percentage in para-carcinoma tissue [26]. Analysis of the microbial composition of the breast revealed that *Streptococcaceae* and Bacillaceae (belonging to Bacillales) were stepwise enhanced in healthy to prediagnostic and postdiagnostic (including adjacent normal and tumour) tissues [38]. The findings suggest a potential signal in early BC diagnosis.

### Most potential targets in BC

As appeared in the three classifications, the related intestinal flora, Ruminococcaceae and *Parabacteroides*, might have a vital role in clinical therapy. Many findings verify that the gut microbiome greatly impacts patients who receive immunotherapy [39]. Additionally, several published papers indicate a differential abundance of Ruminococcaceae in patients who respond to anti-PD-1 immunotherapy [40, 41], as well as of *Parabacteroides distasonis* [42]. *Desulfovibrio*, which showed a causal relationship with total BC and ER-BC, was investigated experimentally in colon cancer [43] and acute gastrointestinal injury [44]. *Sellimonas*, *Adlercreutzia*, and Rikenellaceae were causally related to total BC and ER + BC. However, due to inadequate evidence, including clinical trials and mechanical experiments on BC, further investigation is essential.

Sex hormone dysregulation is acknowledged as one of the main risk factors for development of BC. Female individuals with ER + tumours are considered to benefit from endocrine therapy [45], and female sex hormone levels influence microbiota composition, though the suggestion is bidirectional [46, 47]. A collection of bacteria within the gastrointestinal tract, referred to as the oestrobolome [31], was proven to be involved in production of beta-glucuronidase enzymes and to affect modulation of oestrogen metabolism, circulation, and excretion [48]. Genes encoding ß-glucuronidase are present in several species and bacterial genera in the human gut, including *Edwardsiella*, *Bacteroides*, *Bifidobacterium*, *Collinsella*, *Alistipes*, *Clostridium*, *Citrobacter*, and *Marvinbryantia* [49]. The last genus acts as a potential target for ER + BC. Further exploration is needed.

### Comparison with other studies

Wei et al. published an MR analysis based on five common cancers, in which they highlight that an increased abundance of *Sellimonas* predict a higher risk of ER + BC. This finding is consistent with ours. Another MR study conducted by Long et al. investigated the causal relationship between eight cancers and indicated that *Actinobacteria* and *Bifidobacterium* are risk factors for BC. Both bacterial taxa show significant importance, though they were not included in our findings. Overall, the underlying mechanism of the intestinal microbiome warrants future investigation. In this study, taxa of the gut microbiome were analysed ranging from the genus to phylum level, establishing a conceptual basis for experimentally exploring specific bacterial strains. Furthermore, the results of our study validated the cause-and-effect connection between the gut microbiome and BC and innovatively put forth a viable and achievable therapeutic approach.

### Novel applications

In general, the prognosis of ER-BC is poorer than that of ER + BC. Females diagnosed with ER- are more likely to undergo chemotherapy [50]. Chemotherapies, which mainly involve paclitaxel (PTX), continue to be the most popular and economical treatment, despite the two main drawbacks: toxicity and no specificity in target. Interestingly, the Chinese medicine *Ganoderma lucidum*, when combined with PTX, enhances tumour suppression by restoring the gut microbiota [51]. As some traditional Chinese medicines have been found to regulate the gut microbiota in treating CRC [52], they might provide a novel therapeutic approach for BC.

### Strengths and limitations

This study has some strengths. First, a two-sample MR study was carried out to test the potential causal relationship between the gut microbiota and BC. The genetic variants obtained from a large-scale GWAS led to eliminating the confounding bias caused by the inverse causal problem and supported creditable evidence. Furthermore, gut microbiome taxa were examined from the genus to phylum level, providing a theoretical foundation for experimental investigation of particular bacterial strains. Additionally, the study confirmed the causal relationship of the gut microbiome with BC and further creatively proposes a viable and feasible therapeutic method.

However, there are some significant limitations. First, similar to other MR studies, the effect of weak instrument bias on our findings could not be excluded. Second, the results apply solely to European females because the summary data from the GWAS were subjected to geographical constraints, and more validation experiments to confirm the results presented in this paper are needed. Third, stratified analysis on PR and HER2 was not conducted due to the lack of secondary data. Moreover, p < 5 × 10^-8^ is an accepted cut-off in GWASs; when the GWAS p value was set at 5 × 10^-8^ during the screening process, three types of gut microbiota were identified for each BC subtype. However, the p values were all greater than 0.05, indicating no significant causal relationship. Therefore, we set the GWAS p value threshold as 5 × 10^-5^ in the present study. None of the results in this study achieved a significance level that survived the Bonferroni-corrected threshold, and considering that Bonferroni correction may lead to false-negatives, the results of this study were not subjected to correction. Finally, the gut microbiota might be impacted by environmental or genetic factors that lead to the lower variance explained by genetic instruments.

## Conclusion

In summary, our MR study confirmed that gut microbiota has a causal relationship with BC. It also allowed for explanation of the link between specific bacteria and prognosis for BC with different ER statuses. In addition, some bacteria are regarded as potential targets for clinical treatment. However, further investigations are required to elucidate the underlying mechanism by which the gut microbiota profoundly impacts BC.

### Electronic supplementary material

Below is the link to the electronic supplementary material.

Supplementary Material 1

Supplementary Material 2

Supplementary Material 3

Supplementary Material 4

## Data Availability

Statement. The data used in this study are publicly available. GWAS data. https://www.nature.com/articles/s41588-020-00763-1. MiBioGen consortium. https://mibiogen.gcc.rug.nl/. BC. https://gwas.mrcieu.ac.uk/datasets/ieu-a-1126/. ER- BC. https://gwas.mrcieu.ac.uk/datasets/ieu-a-1128/. ER + BC. https://gwas.mrcieu.ac.uk/datasets/ieu-a-1127/.
